# Prevention of Radiotherapy-Induced Enteropathy by Probiotics (PREP): Double-Blind Randomized Placebo-Controlled Trial

**DOI:** 10.3390/curroncol31100438

**Published:** 2024-10-01

**Authors:** Yoon Young Jo, Yeon Joo Kim, Seung Hae Lee, Young Seok Kim

**Affiliations:** 1 Department of Radiation Oncology, Yeungnam University Medical Center, Daegu 42415, Republic of Korea; eurisko24@yu.ac.kr; 2 Department of Radiation Oncology, Asan Medical Center, University of Ulsan College of Medicine, Seoul 05505, Republic of Korea; kamea1004@naver.com (Y.J.K.);

**Keywords:** radiotherapy, enteropathy, probiotics, *Bacillus licheniformis*

## Abstract

Probiotics are thought to be effective in the treatment of radiation-induced enteropathy (RIE). However, little is known regarding their efficacy in preventing RIE. In this prospective, randomized, double-blinded, placebo-controlled, single-center study, the incidence of grade 2 acute RIE was compared and the safety of probiotics was evaluated. Patients receiving pelvic radiotherapy for a minimum of 40 Gy at the pelvic level were randomized into two groups: (i) a probiotic group receiving *Bacillus licheniformis* from two weeks before radiotherapy until the end and (ii) a control group receiving a placebo with the same schedule. The toxicities of 234 patients were graded according to the Common Terminology Criteria for Adverse Events v5.0. Grade 1 RIE was observed in 65 (56%) of the probiotics group compared with 75 (64%) of the placebo group. Grade 2 RIE occurred in 30 patients (26%) in the probiotics group compared with 26 (22%) in the placebo group, indicating that probiotics failed in their preventive role compared with placebo (*p* = 0.493). Medication adherence rates were good, and no difference was observed between the two arms. These findings suggest that *B. licheniformis* does not significantly prevent RIE.

## 1. Introduction

Radiotherapy (RT) plays an important role in the treatment of tumors in the pelvis, used as either definitive or adjuvant treatment. However, gastrointestinal side effects are common in patients after pelvic RT, with over 70% estimated to develop acute symptoms, approximately 50% of which will have chronic effects affecting their quality of life [1,2]. With an increasing cohort of cancer survivors, efforts to prevent the adverse effects of cancer therapy are crucial. Typical symptoms of radiation-induced enteropathy (RIE) include diarrhea, nausea, vomiting, bloating, indigestion, and abdominal pain. Irradiation of the bowels leads to cell death in rapidly proliferating crypt epithelium and triggers a prolonged inflammatory reaction in the lamina propria [3]. Additionally, radiation alters the bacterial flora, increases the vascular permeability, and affects intestinal motility [4]. However, there are few effective prophylactic agents for RIE.

Probiotics are thought to restore bowel microflora to optimal levels by stimulating mucosal barrier function [5]. Therefore, probiotics could be used to achieve a more balanced microflora during pelvic RT. Several studies [3,6,7,8,9,10,11] have evaluated the effectiveness of probiotics in patients with radiation-induced diarrhea (RID). In addition, several meta-analyses [12,13,14] have suggested that probiotics may offer benefits in the management of RID. However, most studies included small numbers of patients with no information about concomitant chemotherapy. Only two studies started administrating patients on probiotics one week before the start of RT. Most importantly, the RT technique used in this study was a two- or three-dimensional technique, and the criteria used to determine RID also differed.

*Bacillus licheniformis* is a Gram-positive bacterium and can be found in raw milk, buttermilk powder, and pea product as spoiling bacteria. Its use as a probiotic has increased in recent years. The intake of *B. licheniformis* has been reported to be associated with modulation and growth of the gut microbiota, as well as anti-inflammatory effects [15]. However, there is limited information on this strain as a prophylactic use or treatment for RIE. Only one study recently demonstrated that the prophylactic intake of *B. licheniformis* alleviated RT-related gastrointestinal symptoms in pediatric patients with central nervous system tumors [16].

This double-blinded, randomized controlled study was conducted to evaluate whether the prophylactic intake of *B. licheniformis* lead to the prevention of RT-induced enteropathy. To our knowledge, this is the first study to use *B. licheniformis* in pelvic RT settings. The primary endpoint was the incidence of acute RIE, and the secondary endpoint was toxicities resulted from probiotics.

## 2. Materials and Methods

This was a prospective, randomized, double-blinded, placebo-controlled, single-center study. The study was approved by the ethics committee of Asan Medical Center (IRB no. S2019-0696). The study was conducted in accordance with Good Clinical Practice and the Declaration of Helsinki. All patients gave written informed consent to participate in this study. A precise study protocol, including sample size estimation, was available in a previously published protocol review and was registered at ClinicalTrials.gov (accessed on 7 June 2019) (NCT03978949) [17].

The patients included were over 20 years old with an Eastern Cooperative Oncology Group performance status < 2. They had histologically proven malignancies of pelvic cancer and were to receive RT treatment for a minimum of 40 Gy at the pelvic level, with or without chemotherapy. Patients with any medical problems or unexpected probiotics-related toxicities, defined as grade (G) ≥ 3 toxicities based on the Common Terminology Criteria for Adverse Events (CTCAE) v5.0, occurring during treatment were excluded. After stratification by gender, the patients were randomly allocated 1:1 to the probiotics or placebo group using blocked randomization. The block size and randomization sequence varied. The randomization sequence was generated by a central web-based computer to conceal allocation from the primary investigator who enrolled the participants. Neither participants nor physicians were aware of their group assignments. The study flow of the PREP trial is shown in Supplementary Table S1. The study drug is commercially manufactured by Binex (Korea). The manufacturing process is well controlled and validated through quality control testing and is approved by the Korean Food and Drug Administration. One capsule (250 mg) contains 250 million colony-forming units of B. licheniformis which was extracted and separated from pea product (soybean paste). When adding 0.1 g of *B. licheniformis* to peptone KNO3 medium and incubating it at 40 °C for 24 h, it will become turbid and sediment will form. After smearing this culture medium on glucose mineral base agar, it was incubated at 37 °C for 24 h, and mucous colonies in the form of red circular protruding colonies were formed. These colonies were smeared onto nutrient agar to isolate it and use it for inoculation. A total of 0.1 g of this was suspended in 100 mL of 0.1% peptone aqueous solution and then diluted 10 times. An amount of 1 mL of diluted solution was added to a Petri dish, then 10 mL of TSA medium was added, it was mixed well, left to solidify completely at room temperature, and incubated at 37 °C for 24 h. Patients in the probiotics group took two investigational capsules three times daily, starting 2 weeks before RT and continuing every day during RT. Patients in the placebo group received placebo pills according to the same schedule. Patients were required to return their bottles of study medication (probiotics or placebo) weekly, and the number of capsules returned was documented. Patients who took 80–100% of the medication were considered “good”, 60–79% were considered “fair”, and <59% were considered “poor”.

Enhanced computed tomography (CT) simulation for pelvic RT was performed in all patients. Clinical target volume (CTV) included pelvic regional nodal areas based on the Asan Medical Center protocols for each tumor type. The planning target volume (PTV) was created with 3–10 mm expansion in all directions to the CTV. Organs at risk, including the small and large bowel, bladder, rectum, and femoral head, were delineated. All patients received intensity-modulated radiotherapy (IMRT) to the pelvis with daily doses between 2.0 and 2.7 Gy per fraction, 5 days per week, for a total dose of 44–72.6 Gy. Cone-beam CT was used for image guidance during each treatment. Weekly cisplatin 40 mg/m^2^ was the most commonly used regimen for gynecologic and genitourinary cancers.

Patients were evaluated weekly during the scheduled RT and then 3 months after the completion of RT to evaluate the incidence of acute RIE according to CTCAE v5.0. The RIE category was abdominal distension, abdominal pain, belching, bloating, constipation, diarrhea, dyspepsia, flatulence, ileus, nausea, vomiting, and other gastrointestinal symptoms. Furthermore, to demonstrate the safe use of probiotics in patients, adverse events or adverse drug reactions were recorded.

To estimate the sample size, two proportion tests in Power Analysis and Sample Size Software 2018 (NCSS, LLC. Kaysville, UT, USA) were utilized. Assuming 30% of the patients in the placebo group and 15% in the probiotics group would experience grade ≥2 acute toxicity, 118 patients per group were required to demonstrate a significant effect based on an alpha of 0.05 and a beta of 0.2. Considering a 5% dropout rate, 124 patients per group were required. Acute toxicities were analyzed on an intention-to-treat basis, with categorical variables in the two groups compared using the chi-square or Fisher’s exact test.

## 3. Results

A total of 275 patients were recruited from June 2019 to May 2021 and randomly assigned to the probiotics (n = 138) or placebo groups (n = 137). Of these, 41 patients dropped out, mostly due to withdrawal of consent (n = 30). None were excluded due to grade ≥ 3 toxicity. Ultimately, a total of 234 patients were included in the analysis. The general characteristics of the patients and diseases are shown in Table 1. There were no statistical differences between the probiotics and placebo groups.

The incidence of acute RIE is shown in Table 2. Grade 1 RIE occurred in 65 patients (65%) in the probiotics group compared with 75 patients (64%) in the placebo group. Overall, there was no significant difference in RIE (*p* = 0.496). Grade 2 RIE occurred in 30 patients (26%) in the probiotics group compared with 26 patients (22%) in the placebo group. Therefore, there was no significant difference between the incidence of grade 2 RIE (*p* = 0.493), and no grade ≥3 RIE toxicity was observed in the study cohort.

The medication adherence rates were generally good (80–100%), and no differences were observed between the probiotics (75%) and placebo (74%) groups (*p* = 0.437). A poor adherence rate was reported at 11% in the probiotics group and 8% in the placebogroup (Table 3). The intake of probiotics was well-tolerated with low toxicities, with 1% exhibiting grade 2(G2) and 8% exhibiting grade 1 (G1) toxicities, which were identical to those in the placebo group (*p* = 0.420). No septicemia was recorded (Table 4).

## 4. Discussion

This study did not demonstrate the efficacy of probiotics in preventing RIE in patients receiving pelvic RT. Although the incidence of RIE was 84%, most patients had grade 1 RIE, and only about 20% of patients in both arms experienced G2 acute RIE. The probiotics adherence rates were generally good, and the intake of probiotics was well-tolerated. No septicemia was recorded, and no other medicinal side effects of G2 or higher occurred except in one patient.

There are several potential reasons for the negative results in this study. First, the overall incidence of G2 RIE was lower than expected, owing to our cohort’s 100% utilization of IMRT. We hypothesized that 30% of patients in the placebo group would experience ≥G2 acute toxicities; however, only approximately 20% of patients reported G2 RIE in both groups. The characteristics of previous randomized controlled trials and our study results are shown in Supplementary Table S1. Among six RCTs, except for two studies that did not specify the radiation technique, four studies utilized two- or three-dimensional conventional techniques other than IMRT. Therefore, the results of RID were much higher than those of the present study, ranging from 45 to 70%. Considering the current IMRT era, future studies in the larger radiation field and higher radiation dose settings, such as extended field RT and/or definitive treatment, would be appropriate to evaluate the true value of probiotics in preventing RIE. Second, in the present study, the probiotics were not adequate for G1 RIE. A previous study concluded that probiotics appear more effective or only effective in a higher grade of diarrhea, with significant results obtained in subgroups of ≥G2. However, the incidence of G1 diarrhea in the probiotic group was higher than in the placebo group [12]. Third, probiotics needed more time to prevent RIE effectively. Demers et al. [9] reported less diarrhea at 60 days, indicating that the benefit of the probiotics began at the end of the treatment or shortly thereafter. Likewise, Kondo et al. [18] reported that positive effects seem to appear in the fifth to eighth week after the probiotic intake of a specific strain. However, starting the probiotics over one month before RT in real-world is not practical, since we were often unable to delay the patient’s treatment. Lastly, differences were also observed in the strain of probiotics used. Another meaningful difference between our study and those that have reported positive results is the use of multiple bacterial strains. Delia et al. [6] suggested that using several selected strains could enhance the competitive interaction with the intestinal flora. However, the evidence for this remains limited.

Recently, fecal microbiota transplantation, which involves the transfer of a fecal suspension from healthy donors into the gastrointestinal tract of individuals with bowel diseases, has emerged as another potential treatment for RIE [19]. Fecal microbiota transplants comprise bacterial flora extracted from normal feces, fresh stools, or frozen fecal capsules. A pilot study demonstrated both its safety and feasibility [20]. In addition, advances in metagenomics made it possible to identify the gut microbiome at strain level, while 16S rRNA sequencing only identifies the bacterial level [21]. Thus, further studies may provide insights into additional bacterial strains for the prevention and treatment of RIE.

This study has several limitations. We overestimated RIE incidence in the IMRT setting. Consequently, adequate sample size calculations were not achieved. At the time of the present study, grade 2 or higher gastrointestinal toxicities from the previous reports were approximately 30% [22,23]. There may be an argument regarding the strain composition and timing of probiotic intake chosen in the current study. However, an attempt was made to establish a protocol adaptive to real-world practice. Finally, we did not perform nutritional interventions based on the patients’ symptoms, food habits, or requirements, which may influence digestive symptomatology. Nonetheless, this study indicates that single-strain *B. licheniformis* has a limited effect on preventing RIE when administered 2 weeks before the start of pelvic IMRT.

## 5. Conclusions

This study suggests that probiotics containing *B. licheniformis* 250 million colony-forming units do not significantly prevent RIE incidence despite their safety. Further studies will be needed to identify the ideal probiotic composition; ideal high-risk group for developing RIE, for example, a larger RT field; and ideal intake time of probiotics to determine the precise effects of probiotics in the treatment and prevention of RIE.

## Figures and Tables

**Table 1:** Baseline characteristics of the patients and tumors.

Characteristic	Total Patients n = 234 (%)	Probiotics n = 116 (%)	Placebo n = 118 (%)	*p*-Value
Age, year median (range)	64 (28–85)	64 (28–84)	64 (36–85)	0.486
Gender				
Male	143 (61.1)	72 (62.1)	71 (60.2)	0.766
Female	91 (38.9)	44 (37.9)	47 (39.8)	
BMI, kg/m^2^ median (range)	25.1 (18.3–38.1)	24.8 (19.1–38.1)	25.2 (18.3–34.6)	0.782
Comorbidity				
GI comorbidity	12 (5.1)	7 (6.0)	5 (4.2)	0.533
None	222 (94.9)	109 (94.0)	113 (95.8)	
Cancer				
Primary cancer				
Prostate cancer	132 (56.4)	62 (53.4)	70 (59.3)	0.378
Cervix cancer	54 (23.1)	30 (25.9)	24 (20.3)	
Endometrial cancer	31 (13.2)	13 (11.2)	18 (15.2)	
Bladder cancer	11 (4.7)	8 (6.9)	3 (2.5)	
Others	6 (2.6)	3 (2.6)	3 (2.5)	
Use of chemotherapy				
Received	100 (42.7)	51 (44.0)	49 (41.5)	0.706
None	134 (57.3)	65 (56.0)	69 (58.5)	
Operation				
Received	132 (57.3)	72 (62.0)	62 (52.5)	0.141
Not received	100 (42.7)	44 (38.0)	56 (47.5)	
Use of ADT				
Received	116 (49.6)	53 (45.7)	63 (53.4)	0.239
None	118 (50.4)	63 (54.3)	55 (46.6)	
Aim of RT				
Definitive	104 (44.4)	45 (38.8)	59 (50.0)	0.204
Postoperative	49 (20.9)	28 (24.1)	21 (17.8)	
Salvage	81 (34.6)	43 (37.1)	38 (32.2)	
RT duration, days median (range)	45.0 (23.0–66.0)	44.0 (23.0–62.0)	45.0 (23.0–66.0)	0.307
RT dose, Gy median (range)	66.0 (44.0–72.6)	66.0 (44.0–72.6)	66.0 (46.0–72.6)	0.296
Fraction size, Gy median (range)	2.2 (2.0–2.7)	2.2 (2.0–2.5)	2.2 (2.0–2.7)	0.286
RT field				
Pelvis	222 (94.9)	112 (96.6)	110 (93.2)	0.248
Abdominopelvic	12 (5.1)	4 (3.4)	8 (6.8)	

ADT, androgen deprivation therapy; BMI, body mass index; GI, gastrointestinal; RT, radiotherapy.

**Table 2:** Incidence of acute radiation-induced enteropathy.

Variable	Total Patients n = 234 (%)	Probiotics n = 116 (%)	Placebo n = 118 (%)	*p*-Value
None	38 (16)	21 (18)	17 (14)	0.496
Grade 1	140 (60)	65 (56)	75 (64)	
Grade 2	56 (24)	30 (26)	26 (22)	
Grade 3–5	0 (0)	0 (0)	0 (0)	
Total	234 (100)	116 (100)	118 (100)	

**Table 3:** Medication adherence rates *.

Variable	Total Patients n = 234 (%)	Probiotics n = 116 (%)	Placebo n = 118 (%)	*p*-Value
Good (80–100%)	174 (74.4)	87 (75.0)	87 (73.7)	0.437
Fair (60–79%)	38 (16.2)	16 (13.8)	22 (18.6)	
Poor (0–59%)	22 (9.4)	13 (11.2)	9 (7.6)	
Total	234 (100)	116 (100)	118 (100)	

* Probiotics or placebo capsules.

**Table 4:** Probiotics/placebo capsules induced acute side effects *.

Variable	Total Patients n = 234 (%)	Probiotics n = 116 (%)	Placebo n = 118 (%)	*p*-Value
None	210 (89.7)	107 (92.2)	103 (87.3)	0.420
Grade 1	22 (9.4)	8 (6.9)	14 (11.9)	
Grade 2	2 (0.9)	1 (0.9)	1 (0.8)	
Grade 3–5	0(0)	0(0)	0(0)	
Total	234 (100)	116 (100)	118 (100)	

* Measured on the first day of RT after taking the drug 2 weeks before the start of RT.

## Data Availability

All data generated or analyzed during this study are included in this article. Further inquiries can be directed to the corresponding author.

## Supplementary Materials

The following supporting information can be downloaded at: https://www.mdpi.com/article/10.3390/curroncol31100438/s1. Table S1: Characteristics of previous randomized controlled trials studying the effects of probiotics on radiation-induced enteropathy.
