# Supplementary material for: Prevention of Radiotherapy-Induced Enteropathy by Probiotics (PREP): Double-Blind Randomized Placebo-Controlled Trial
Source: Curr Oncol. 2024 Oct 1;31(10):5889–95. doi: 10.3390/curroncol31100438 (PMC11506454; doi:10.3390/curroncol31100438)
Supplement: Supplementary file 1 [file curroncol-31-00438-s001.zip › curroncol-3199467-supplementary.pdf]

Supplementary Table S1. Characteristics of previous randomized controlled trials studying the effects of probiotics on radiation-induced enteropathy

First author (year, country)	Patient no. (placebo/probiotics)	Primary tumor	RT aim	Total RT dose (Gy)	RT technique	Chemotherapy	Timing	Species	Toxicity type	Acute toxicity placebo/probiotics (%)	Result
Delia (2007, Italy) [6]	239/243	Cervix, rectum, sigmoid	PORT	60–70	2D	NA	During RT	*L. casei, L. acidophilus, L. rhamnosus, L. bulgaricus, B. vreve, B. longum, S. thermophilus*	Diarrhea	Any grade 52/32	SS
Giralt (2008, Spain) [7]	41/44	Cervix, endometrium	PORT	40–50	3D	Cisplatin for cervix	1 week before RT	*L. casei*	Grade ≥2 diarrhea	Grade ≥2 59/68	NS
Chitapanarux (2010, Thailand) [8]	31/32	Cervix	NA	40–56	2D/3D	Cisplatin	1 week before RT	*L. acidophilus B. bifidium*	Diarrhea	Grade 2–3 45/9	SS
Demers (2014, Canada) [9]	86/140	GY, rectum, prostate	NA	40–50.4	NA	Cisplatin for cervix, Xeloda for rectum	During RT	*L. acidophilus B. longum*	Diarrhea	Moderate-to-severe 67/64	NS
Tehrani (2016, Iran) [10]	24/43	GY, GU, rectum, bone sarcoma	NA	40–50	NA	NA	1 week before RT	*L. casei, L. acidophilus, L. rhamnosus, L. bulgaricus, B. vreve, B. longum, S. thermophilus*	Diarrhea	Moderate-to-severe 71/19-32	SS
Linn (2018, Myanmar) [11]	28/26	Cervix	NA	50	2D	NA	Druing RT	*L. acidophilus, B. animalis*	Diarrhea	Mild-to-moderate 88/54	SS
Current (2021, Korea)	118/118	GY, GU	PORT, Definitive	44.0–72.6	IMRT	Cisplatin for cervix	2 weeks before RT	*B. licheniformis*	Acute GI toxicity	G2 RIE 22/21	NS

RT, radiotherapy; 2D, two-dimensional; 3D, three-dimensional; *B, bifidobacterium*; CTCAE, Common Terminology Criteria for Adverse Events; CTC of NCI, common toxicity criteria of National Cancer Institute; IMRT, intensity modulated radiotherapy; *L, lactobacillus*; NA, not available; NS, not significant; PORT, postoperative radiotherapy; SS, statistically significant; *S, streptococcus*; WHO, world health organization
